# Effects of Artificial Tooth Brushing and Hydrothermal Aging on the Mechanical Properties and Color Stability of Dental 3D Printed and CAD/CAM Materials

**DOI:** 10.3390/ma14206207

**Published:** 2021-10-19

**Authors:** Na-Eun Nam, Seung-Ho Shin, Jung-Hwa Lim, June-Sung Shim, Jong-Eun Kim

**Affiliations:** 1 BK21 FOUR Project, Department of Prosthodontics, Yonsei University College of Dentistry, Yonsei-ro 50-1, Seodaemun-gu, Seoul 03722, Korea; jennynam5703@prostholabs.com (N.-E.N.); shin506@prostholabs.com (S.-H.S.); erin0313@prostholabs.com (J.-H.L.); 2 Department of Prosthodontics, Yonsei University College of Dentistry, Yonsei-ro 50-1, Seodaemun-gu, Seoul 03722, Korea; jfshim@yuhs.ac

**Keywords:** 3D printing, additive manufacture, CAD/CAM, artificial toothbrushing, mechanical property, dental materials

## Abstract

This study analyzed the surface roughness and waviness, Vickers hardness (VHN), and color changes of six types of 3D printed resins and computer-aided design/computer-aided manufacturing (CAD/CAM) materials after artificial toothbrushing. The average surface roughness height (Ra) change of Formlabs denture teeth A2 resin (FMLB) was not significant between after artificial toothbrushing (0.17 ± 0.02 μm and 0.17 ± 0.05 μm, respectively; mean ± standard deviation). However, the Ra value increased significantly in all remaining groups. Regarding waviness, polymethylmethacrylate (PMMA) had the largest increases in average waviness height (Wa) and maximum surface waviness height (Wz) between, before (0.43 ± 0.23 μm and 0.08 ± 0.02 μm), and after (8.67 ± 4.03 μm, 1.30 ± 0.58 μm) toothbrushing. There were no significant changes in Wa for Formlabs denture teeth A2 resin (FMLB) and NextDent C&B (NXT). After artificial toothbrushing, the dispersed-filler composite (DFC) group had the largest color difference (ΔE, of 2.4 ± 0.9), and the remaining materials had smaller changes than the clinical acceptance threshold of ΔE = 2.25. The VHN of FMLB and NXT were 9.1 ± 0.4 and 15.5 ± 0.4, respectively, and were not affected by artificial toothbrushing. The flexural strengths of the 3D printed materials were 139.4 ± 40.5 MPa and 163.9 ± 14.0 MPa for FMLB and NXT, respectively, which were similar to those of the polycarbonate and PMMA groups (155.2 ± 23.6 MPa and 108.0 ± 8.1 MPa, respectively). This study found that the evaluated 3D printed materials had mechanical and optical properties comparable to those of CAD/CAM materials and were stable even after artificial toothbrushing and hydrothermal aging.

## 1. Introduction

Computer-aided design/computer-aided manufacturing (CAD/CAM) systems have rapidly developed recently in dentistry, allowing the use of various types of digital equipment and materials when fabricating dental restorations [1]. Digital impression data obtained through a model or oral scanner using the CAD/CAM system can be used for prosthesis design via CAD software. The completed design data are processed by milling equipment or a 3D printer to produce dental restorations [2]. A digital-based prosthesis fabrication method reduces errors that can occur in traditional fabrication methods and improves efficiency [3,4], which has led to it being applied in various treatment fields to replace conventional methods. Digital manufacturing includes subtractive and additive manufacturing, which involving computer-aided milling and using 3D printers, respectively [5,6]. Milling technology, where restorations are manufactured by cutting commercially available blocks through a high-temperature and high-pressure polymerization process, produces restorations comparable to those manufactured by conventional techniques in terms of mechanical performance, biological properties, and accuracy [7,8]. However, since the prosthesis is produced using a subtractive method, a considerable consumption of material and milling burs occur, and is it difficult to manufacture complex shapes [9,10]. Therefore, the interest in using 3D printing as a substitute for milling has increased recently for the manufacturing of dental prostheses [11].

The American Society for Testing and Materials defines the additive manufacturing method of 3D printing as “a process of combining materials to create objects from 3D model data, rather than a subtractive manufacturing methodology” [5]. This method has been effectively applied for rapid prototyping during the production of highly customized models [12]. In dentistry, it is applied to implant surgery guides, dental models, and occlusal splint production, and various dental 3D printed materials are also being developed [13]. Manufacturing dental prostheses using additive 3D printing technology reduces material waste and allows the processing of multiple prostheses simultaneously [5,14]. Prostheses fabricated using 3D printing were found in a recent study to have similar precision to those fabricated using milling or conventional techniques [15,16,17,18,19]. Due to the aforementioned advantages, 3D printed resin materials can be an alternative to CAD/CAM milling materials for dental applications.

Crown bridge resins must be strong enough to withstand the large chewing forces in the oral cavity, and it must exhibit abrasion resistance and color stability during long-term use [20,21]. Roughness values above 0.2 μm have been reported to be related to an increase in bacterial retention and plaque accumulation [22], which can lead to a risk of gingival and periodontal inflammation [23,24]. Also, an increase in roughness can cause discoloration of the restoration and thus impair their esthetic appearance [25]. Brushing with a toothbrush and abrasive toothpaste plays an important role in the changes of surface roughness with restorative materials [26]. The wear resistance of a material affects its surface roughness, and increased roughness may indicate a deteriorated material [27]. Therefore, laboratory wear tests such as artificial brushing can help determine the longevity of dental materials [26,28,29]. Previous studies of artificial toothbrushing have evaluated and identified the differences in materials [1,30,31]. However, no previous studies that we know of have evaluated changes in the properties of dental 3D printed materials after artificial toothbrushing. Therefore, it is unknown whether 3D printed resin materials have mechanical and optical stability that can replace current CAD/CAM milling materials. Instead, previous studies analyzed the abrasion resistance and surface properties of the material after a chewing simulation to evaluate the mechanical properties of 3D printed resin [11]. That study involved only a short simulation period equivalent to about 1 month in the oral cavity, and only analyzed the volume loss of the material. Not identifying changes in the roughness of materials that could affect microorganism growth was therefore a major limitation of that study. Despite the gradual developments in 3D printing crown bridge materials, studies on the differences between the mechanical properties of different material or changes that occur after their use in the oral cavity over long periods have been insufficient.

The purpose of this study was therefore to compare the surface roughness, surface waviness, Vickers hardness (VHN), color change, and intergroup VHN change after artificial toothbrushing on 3D printed material and CAD/CAM blocks. The study was also designed to determine the difference between groups in flexural strength and the changes after hydrothermal aging treatment. The first null hypothesis of this study was that artificial toothbrushing does not affect the surface roughness, surface waviness, VHN, or color of the materials, and that there is no VHN difference between groups. The second null hypothesis was that flexural strength does not differ between groups and hydrothermal aging has no effect.

## 2. Materials and Methods

**Figure 1:**
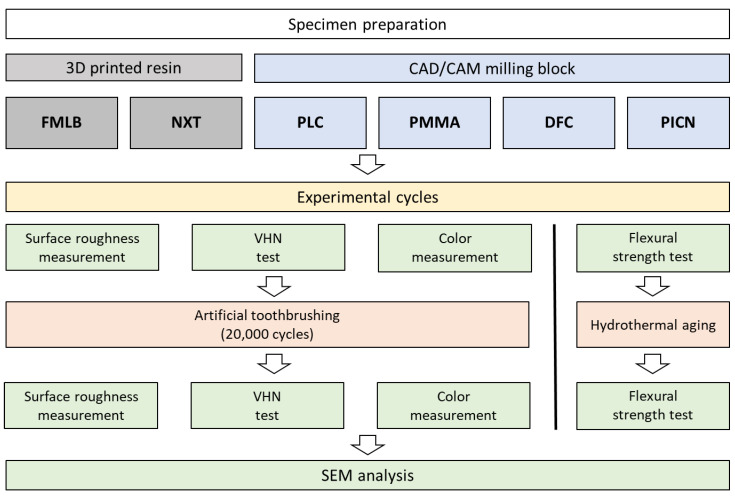
Overall experimental workflow showing the materials used and experimental cycles. Material names are coded as follows: FMLB (Formlabs denture teeth A2 resin), NXT (NextDent C&B), PLC (polycarbonate), PMMA (polymethylmethacrylate), DFC (dispersed-filler composite), and PICN (polymer infiltrated ceramic network).

The overall experimental workflow is summarized in Figure 1.

The following six materials were used in this study and the material names are coded (Table 1): Formlabs denture teeth A2 resin (FMLB) (Formlabs, Sommerville, MA, USA), NextDent C&B (NextDent, Soesterburg, the Netherlands), polycarbonate (PLC) (polycarbonate block, Line Dental Lab, Seoul, Korea), polymethylmethacrylate (VIPI BLOCK, VIPI, São Paulo, Brazil), dispersed-filler composite (DFC) (MAZIC Duro, Vericom, Chuncheon, Korea), and polymer-infiltrated ceramic network (PICN) (VITA ENAMIC, VITA Zahnfabrik, Bad Säckingen, Germany).

### 2.1. Specimen Preparation

A precision cutting machine cut (ADM-6S, Okamoto, Tokyo, Japan) ∅98, 18 mm discs made of three CAD/CAM materials (PLC, MZD, and VTE), which were then trimmed using a diamond wheel (#400) and slurry (grit sizes of 6 μm, 3 μm, and 1 μm). For artificial toothbrushing, a surface roughness, surface waviness, VHN, and color measurement test specimen was designed with a length of 10 mm, a width of 18 mm, and a thickness of 4 mm. With a length of 25 mm, a width of 2 mm, and a thickness of 2 m, a test specimen was designed for the flexural strength test. The cut specimens were washed using deionized water in an ultrasonic cleaner for 3 min, and then air-dried. A 3D Digital Light Processing 3D printer with a 405-nm ultraviolet (UV) light-emitting diode (LED) (NextDent 5100, Vertex-Dental, Soesterberg, Netherlands) printed the NextDent C&B (NXT) specimen. A stereolithography 3D printer with a 405-nm UV LED and a laser power of 250 mW (Form3, Formlabs) was used to print the FMLB specimen. The printed specimens were washed for 10 min in a washing machine (Twin Tornade, Medifive, Seoul, Korea) with 90% isopropyl alcohol. The postcuring process was conducted for 30 min in accordance with the conditions recommended by the manufacturer of the UV postcuring equipment (CureM D102, Sona Global, Seoul, Korea).

### 2.2. Artificial Toothbrushing

Color, surface roughness, surface waviness, and VHN measurements were performed sequentially. All tested specimens were placed in distilled water and stored in an incubator (JISICO, Seoul, Korea) at 37.5 °C for 1 week. Each specimen was subject to 40,000 brushing strokes over 4 h and 44 min at 75 strokes per minute using an artificial toothbrushing device (V-8 cross-brushing machine). Artificial toothbrushing was performed by applying a vertical load of 2.0 N using an FDA-approved toothbrush (Colgate Twister, Colgate-Palmolive, São Paulo, SP, Brazil). A toothpaste slurry was used according to ISO standard [32] by mixing 250 g of toothpaste (Colgate Total, Colgate-Palmolive) with a relative dentin abrasivity of 70 with 1 L of distilled water, with the slurry being replaced every 5000 cycles. The specimen was washed with water and dried after the brushing strokes were completed.

### 2.3. Surface Roughness and Surface Waviness Measurements

**Figure 2:**
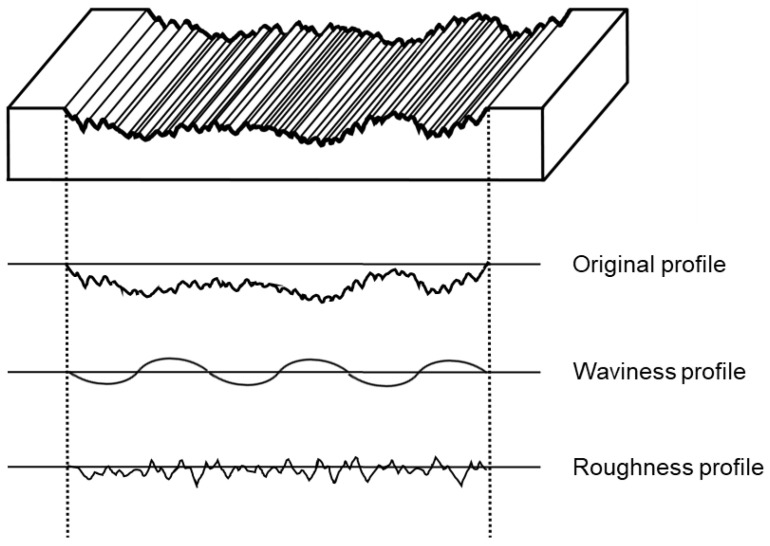
Surface roughness and surface waviness.

The surface of the specimens before and after artificial toothbrushing were measured (Figure 2) according to ISO Standard 4287: the average roughness height (Ra), maximum surface roughness height Rz, average waviness height (Wa), and maximum surface waviness height (Wz) [33]. A stylus profilometer (Bruker Dektak XT, Bruker, Germany) was applied over 5.6 mm with a cutoff value of 0.8 mm and a stylus speed of 0.5 mm per second. Surface roughness and waviness values were calculated based on these data.

### 2.4. VHN Test

VHN values were measured using microhardness indentation equipment (HMV-G, Shimadzu Corporation, Tokyo, Japan) before and after artificial toothbrushing. Each of the six groups used 15 specimens each (n = 75). Indentations were made three times on specimens with dimensions of 10 mm × 8 mm × 4 mm, and the VHN was measured. The respective lengths of two diagonal lines appearing in the indentation were measured to calculate VHN. Indentation was performed by applying a force of 1.961 N for 15 s to obtain VHN values, and the average VHN of the three measurements was used. VHN was calculated using the following formula (1):

(1) $$\mathrm{HV}=1.854\left(F/D^{2}\right)$$

where F is the applied load in newton and D is the area over which the indenter applied the load, measured in square millimeters.

### 2.5. Color Measurement

A colorimeter (Cr321 Chromameter, Minolta, Osaka, Japan) was used before and after artificial toothbrushing to measure the color of the center of the specimen. Each of the six groups used 15 specimens each (n = 75). The color measurement was performed three times on the specimen and the average value was used. Commission Internationale d’Eclairage L* a* b* (CIELab) system: L* (lightness, from 0 [black] to 100 [white]), a* (from a [green] to +a [red]), and b* (from b [blue] to +b [yellow]) was used. The color difference $\Delta E_{00}$ was calculated using the following formula (2):

(2) $$\Delta E_{00}=\sqrt{{\left(\frac{\Delta L}{K_{L}S_{L}}\right)}^{2}+{\left(\frac{\Delta C}{K_{C}S_{C}}\right)}^{2}+{\left(\frac{\Delta H}{K_{H}S_{H}}\right)}^{2}+R_{T}\left(\frac{\Delta C}{K_{C}S_{C}}\right)\left(\frac{\Delta H}{K_{H}S_{H}}\right)}$$

### 2.6. Flexural Strength Test

Fracture testing was performed on 180 prepared specimens (4 mm × 1.2 mm × 18 mm; 30 specimens for each material), each of which was divided into hydrothermal aging and nonaging groups (n = 15 for both hydrothermal aging and nonaging). For the hydrothermal aging specimens, a 2-bar pressure was applied at 121 °C for 5 h using a hydrothermal reactor to simulate the long-term environment within the mouth of a patient. After the completion of treatment, a flexural three-point bending test was performed using a universal testing machine (Instron 8871; Instron Co., Corwood, OH, USA) with a crosshead speed of 1.0 mm/minute and a distance of 10 mm between the support spans, with the following formula used (3):

(3) $$\sigma =3\mathrm{Fl}/2{\mathrm{bh}}^{2}$$

where F was the breaking load in newton, l is the span-to-span distance in millimeters, b is the specimen width in millimeters, h is the specimen height in millimeters, and d is the deflection in millimeters.

### 2.7. Scanning Electron Microscopy (SEM) Observations

The surface of each specimen was observed using a scanning electron microscope (7800 F FESEM, JEOL, Peabody, MA, USA) to determine the pattern of changes between, before, and after toothbrush abrasion and the flexural strength test. The surface of the specimen was coated with platinum for 30 s using an osmium target (HPC-1S, Shinku Device, Ibaraki, Japan), and then SEM was performed under an accelerating voltage of 5 kV. SEM micrographs were obtained at 30–1000 times magnification for visual observations.

### 2.8. Statistical Analysis

Statistical analyses were performed using SPSS software (version 25, IBM, Armonk, NY, USA). To determine differences between groups and within groups, one-way ANOVAs were conducted to determine significance, and the means and standard deviations were calculated for each group, with the errors compared and evaluated. A Scheffe’s test was performed at a significance cutoff of α = 0.05. To confirm any change after artificial toothbrushing, a paired-samples *t*-test was performed at a significance cutoff of α = 0.05.

## 3. Results

### 3.1. Surface Roughness and Surface Waviness

**Figure 3:**
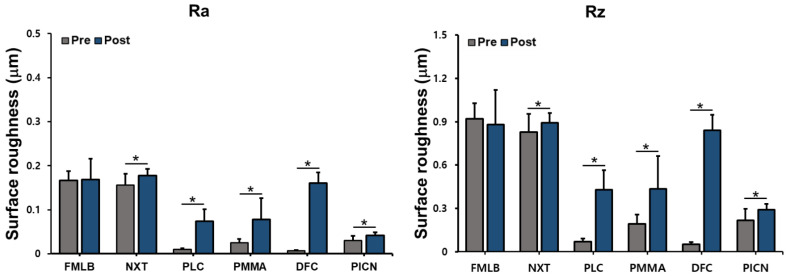
Surface roughness (Ra, Rz; data are mean and standard-deviation values) of six materials pre and post artificial toothbrushing. Asterisks indicate statistically significant differences (*p* < 0.05).

After measuring the Ra and Rz of six materials before and after artificial toothbrushing, a paired-sample *t*-test was performed to identify any difference after toothbrushing (Figure 3 and Table S1). Regarding FMLB, the Ra values before and after artificial brushing were 0.17 ± 0.02 μm and 0.17 ± 0.05 μm, respectively, with no significant difference (*p* = 0.837); the corresponding Rz values were 0.92 ± 0.11 μm and 0.89 ± 0.07 μm (*p* = 0.508). However, in all groups except FMLB, the Ra and Rz values increased significantly after toothbrushing: NXT (Ra; *p* = 0.006, Rz; *p* = 0.028), PLC (Ra; *p* < 0.001, Rz; *p* < 0.001), PMMA (Ra; *p* = 0.002, Rz; *p* = 0.003), DFC (Ra, *p* < 0.001; Rz, *p* < 0.001), and PICN (Ra, *p* = 0.004; Rz, *p* = 0.008).

**Figure 4:**
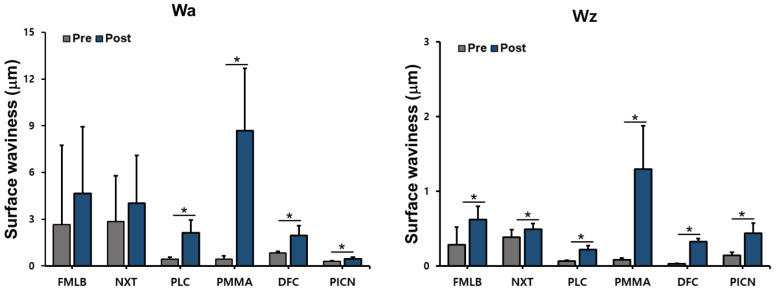
Surface waviness (Wa, Wz; data are mean and standard-deviation values) of six materials pre and post artificial toothbrushing. Asterisks indicate statistically significant differences (*p* < 0.05).

Wa and Wz were measured before and after artificial toothbrushing on six materials, and paired-samples *t*-tests identified any significant difference after artificial toothbrushing for each material (Figure 4 and Table S1). The paired-samples tests suggested that the Wa and Wz values of the posttest significantly increased in all groups except for Wa in FMLB (*p* = 0.309) and NXT (*p* = 0.367): FMLB (Wz, *p* < 0.001), NXT (Wz, *p* = 0.011), PLC (Wa, *p* < 0.001; Wz, *p* < 0.001), PMMA (Wa, *p* < 0.001; Wz, *p* < 0.001), DFC (Wa, *p* < 0.001; Wz, *p* < 0.001), and PICN (Wa, *p* = 0.001; Wz, *p* < 0.001).

### 3.2. VHN Test

**Figure 5:**
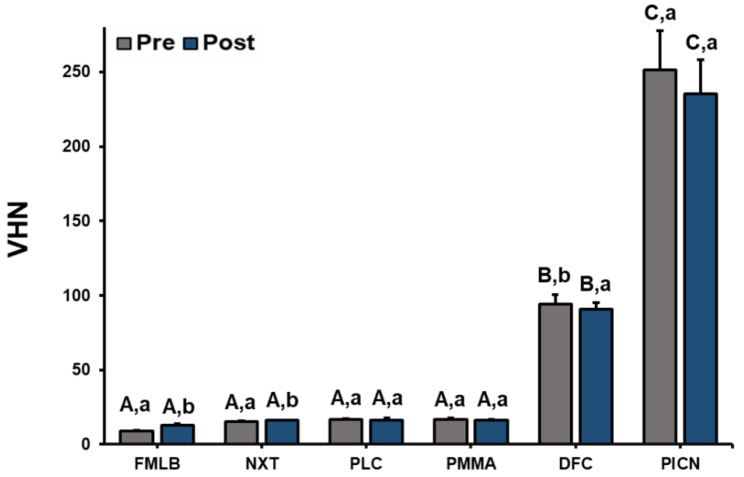
Surface hardness (data are mean and standard-deviation values) of six materials pre- and post-artificial toothbrushing. A significant difference in surface hardness between materials is indicated by different upper-case letters, and a significant difference in surface hardness between pre and post artificial toothbrushing is indicated by different lower-case letters (*p* < 0.05).

To determine the micromechanical properties of all materials, VHN was measured after polishing the surface and after artificial toothbrushing (Figure 5 and Table S2). First, after measuring the VHN of each material, a one-way ANOVA was performed to determine the VHN differences between the materials. These tests indicated significant differences in VHN according to the material both before (F = 1133.177, *p* < 0.001) and after (F = 1314.113, *p* < 0.001) toothbrushing. Performing a Scheffe’s post-test to confirm the difference confirmed that the VHN of DFC was higher than those of FMLB, NXT, PLC, and PMMA, and that the VHN of PICN was the highest both before and after artificial toothbrushing.

A paired-samples *t*-test was performed to determine any significant difference of VHN results after toothbrushing for each material, which revealed no changes between the pre- and posttest results for PLC (*p* = 0.661), PMMA (*p* = 0.152), and PICN (*p* = 0.164), but significant changes for FMLB (*p* < 0.001), NXT (*p* = 0.001), and DFC (*p* = 0.025) appeared. The VHN values of FMLB and NXT increased after artificial toothbrushing from 9.1 ± 0.4 and 15.5 ± 0.4, respectively, to 12.7 ± 1.0 and 16.1 ± 0.3. Regarding DFC, the VHN decreased from 94.1 ± 6.3 before to 90.6 ± 4.5 after artificial toothbrushing.

### 3.3. Color Change

**Figure 6:**
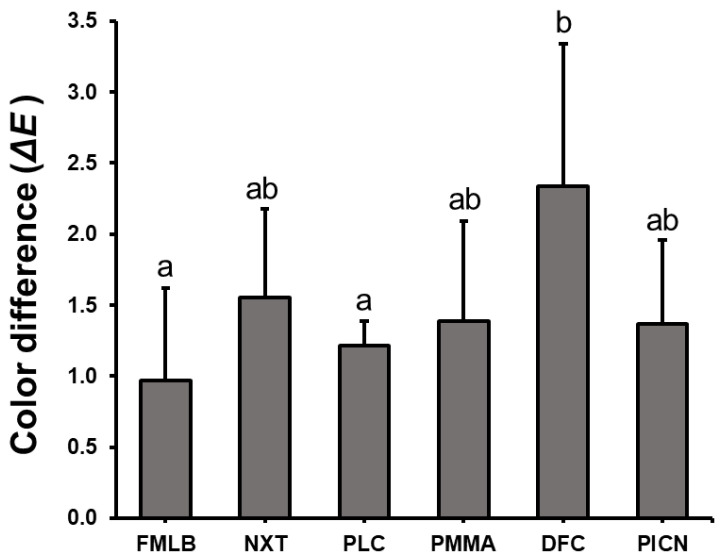
Color differences of six materials after artificial toothbrushing. Significant differences in color change between materials are indicated by different lower-case letters (*p* < 0.05).

The colors of the materials were measured and then compared between, before, and after artificial toothbrushing. One-way ANOVA was performed to identify differences in color changes between materials (Figure 6, Table S3), which revealed a significant difference in color difference between groups (F = 6.095, *p* < 0.001). The color difference between FMLB and PLC was significantly lower than for the other samples, at 1.0 ± 0.6 and 1.3 ± 0.3, respectively. The color differences of NXT, PMMA, and PICN were 1.6 ± 0.6, 1.4 ± 0.9, and 1.6 ± 0.8, respectively, and the color difference of the DFC group was 2.4 ± 0.9, which was significantly larger than those of the other groups. Color differences larger than ΔE = 2.25 were considered clinically observable; the ΔE values of all materials, except for the DFC group, reached this criterion.

### 3.4. Flexural Strength

**Figure 7:**
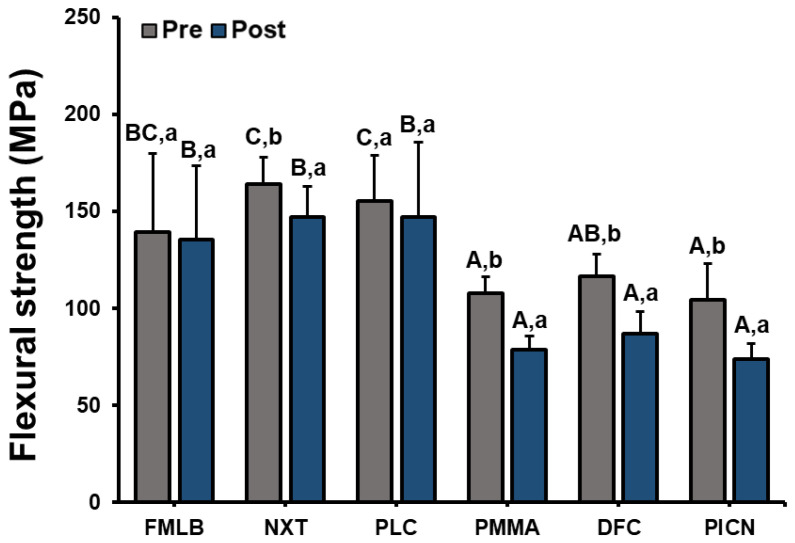
Flexural strength results of six materials pre and post toothbrushing. A significant difference in flexural strength between materials is indicated by different upper-case letters, and a significant difference in flexural strength between pre and post artificial toothbrushing is indicated by different lower-case letters (*p* < 0.05).

A three-point flexural strength test was performed on all materials, and one-way ANOVA was performed to identify any significant difference in flexural strength between the materials. There were significant differences between materials before (F = 19.568, *p* < 0.001) and after (F = 33.183, *p* < 0.001) artificial toothbrushing (Figure 7, Table S4). Scheffe’s post-hoc test indicated that the flexural strengths of PLC and NXT were 163.9 ± 14.0 MPa and 155.2 ± 23.6 MPa, respectively, which were significantly larger than those of the other groups, with the next highest being FMLB at 139.4 ± 40.5 MPa. The flexural strengths of PICN and PMMA were 104.2 ± 19.0 MPa and 108.0 ± 8.1 MPa, respectively, which were significantly smaller than that of DFC (116.4 ± 11.4 MPa). Our analysis indicated a difference between the materials after hydrothermal aging. The flexural strengths of the NXT, PLC, and FMLB groups were 147.2 ± 15.7 MPa, 147.1 ± 38.3 MPa, and 135.5 ± 37.8 MPa, respectively, which were significantly larger than those of DFC (86.9 ± 11.4 MPa), PMMA (78.5 ± 7.3 MPa), and PICN (73.6 ± 8.0 MPa).

Paired-samples *t*-tests were performed to identify any significant difference between the experimental results before and after hydrothermal aging for each material (Figure 7). These tests indicated that all groups except FMLB (*p* = 0.796) were differed significantly after hydrothermal aging: NXT (*p* = 0.018), PLC (*p* = 0.458), PICN (*p* < 0.001), PMMA (*p* < 0.001), and DFC (*p* < 0.001). The PMMA and FMLB groups exhibited the largest and smallest changes in flexural strength, respectively.

### 3.5. SEM Observations

**Figure 8:**
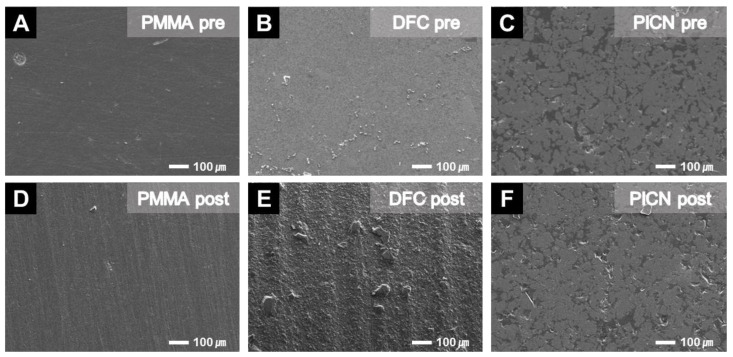
Surface conditions of the specimens, pre- and post-artificial toothbrushing, in SEM images at 1000-times magnification. Surface changes occurred after artificial toothbrushing, with the degree of difference depending on the material. The Rz change in DCF appeared to be the largest, and shallow streaks appeared on the surface of the PMMA specimen. The PICN surface was almost unchanged. (**A**) PMMA pre, (**B**) DFC pre, (**C**) PICN pre, (**D**) PMMA post, (**E**) DFC post, and (**F**) PICN post.

Each material surface was analyzed using SEM before and after toothbrushing (Figure 8). After polishing, the specimen surfaces were generally smooth, but streaks that appeared to be caused by the sliding of the toothbrush were present on the surface after artificial toothbrushing. The surface of the PICN specimen was smooth both before and after toothbrushing, and no change in roughness was evident, which was consistent with the results of the Ra and Rz analysis. On the other hand, the surface of the DFC specimen appeared to be considerably rougher after artificial brushing, and rougher than the surfaces of the PMMA and PICN specimens. This was also consistent with the roughness values of the Ra and Rz analysis.

**Figure 9:**
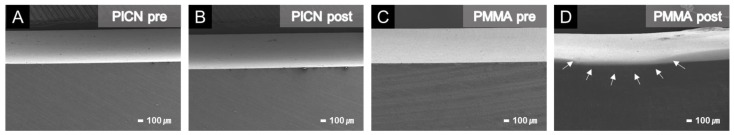
SEM images of cross sections at 35-times magnification. There was no significant change in the surface of PICN between pre and post artificial brushing, whereas PMMA had a concave planar shape after brushing due to abrasion. The arrows in panel D indicate the concave, worn surface of the PMMA specimen. (**A**) PICN pre, (**B**) PICN post, (**C**) PMMA pre, and (**D**) PMMA post.

The cross-sectional view images of the PICN and PMMA specimens before and after artificial toothbrushing (Figure 9) indicate that the PICN specimen had almost no surface damage, whereas the PMMA specimen had a concave shape as the surface wore out. Among the tested materials, the images indicate that the wear appeared to be greatest for the PMMA specimen, which was consistent with the Wa and Wz values obtained in the surface waviness analysis.

**Figure 10:**
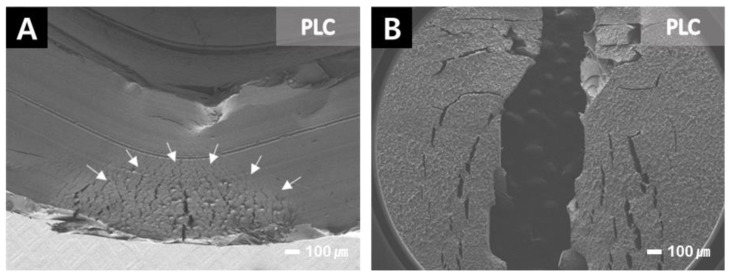
SEM images of the PLC specimen after its flexural strength test at 30-times magnification. The PLC specimens were high elasticity did not break easily. Many fatigue crack lines were observed on the fracture surface. The arrows in panel indicate the fatigue crack lines. (**A**) Unbroken PLC specimen after fracture test, and (**B**) top side of a broken specimen after the flexural strength test.

**Figure 11:**
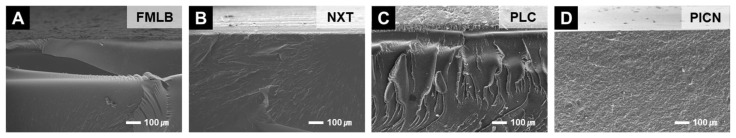
SEM images of the fracture surface of broken specimens after their flexural strength tests at 100-times magnification. Different fracture surface shapes appeared depending on the material properties. A cleaner fracture surface was observed on the relatively brittle PICN specimen when compared with the FMLB and NXT specimens. Fatigue crack lines were often observed at the fracture surface of PLC samples with high elasticity. (**A**) FMLB, (**B**) NXT, (**C**) PLC, and (**D**) PICN.

Most PLC specimens did not break in the flexural strength test (Figure 10). Fatigue crack lines were observed on the bent surface of the PLC specimen. Similarly, the PICN had a relatively clean fractured surface when compared with the cross section of the fractured PLC specimen, and a clear fatigue crack line appeared on the fracture surface of the PLC specimen (Figure 11).

## 4. Discussion

This study evaluated the surface roughness, surface waviness, VHN, and color change after artificial toothbrushing on 3D printed materials and CAD/CAM blocks. Differences were evaluated between groups in flexural strength between, before, and after hydrothermal aging treatment. Our experiments also identified changes in surface roughness, surface waviness, VHN, and color after artificial toothbrushing, and also a difference in VHN between materials. The flexural strength of each material was different, and tended to decrease after hydrothermal aging treatment. Therefore, the null hypotheses of this study were rejected. 

This study applied 20,000 cycles of artificial toothbrushing to simulate 7 to 10 years of material use [34]. Also, The ISO standards were used to provide comparative results of 3D printed resin, which has no experimental results under similar conditions to previously tested milling materials. Three parameters were used to evaluate changes in surface roughness and waviness that appeared thereafter. Ra and Rz, measured using a contact stylus, is one of the important vertical parameters representing the depth of scratches present on the material surface, and is often used to quantify worn cross sections [30]. Vertical volume loss was also quantitatively evaluated by analyzing Wa and Wz waviness, and SEM imaging analysis provided an overall understanding of the material surface morphology changes [30]. These experiments indicated that the roughness values of all groups significantly increased after toothbrushing. FMLB, NXT, and DFC specimens showed significantly higher roughness, whereas PICN was indicated as having the smallest changes in surface roughness and waviness. These differences between materials may be due to how the microstructure of PICN provides excellent fatigue resistance via a reinforcing effect [27,29], and is composed of a double interpenetrating network created by the PICN technology [35,36].

Koizumi et al. [30,31] performed brushing on a CAD/CAM block, and the difference in Rz between specimens was compared after using each material. This revealed that Lava Ultimate, a composite resin with a composition similar to that of DFC, had a significantly larger Rz than did PICN, which was consistent with our results. This was also consistent with Kamonkhantikul et al. [30] finding that PICN was significantly less rough than PMMA after 20,000 toothbrushing cycles. An Ra of 0.25–0.5 μm can be detected by the patient’s tongue [25] and a roughness value larger than 0.2 μm can affect bacterial growth [1]. Although the increase in the surface roughness after toothbrushing was statistically significant, it was lower than the clinically acceptable value of 0.2 μm, indicating that it would be stable for medium- and long-term use. The evaluated 3D printed material was also indicated to have surface roughness characteristics comparable to those of the existing CAD/CAM material. To measure surface waviness, the stylus moved sideways perpendicular to the worn surface of the specimen, which allowed the vertical loss of the specimen to be quantified [25]. After identifying the change in surface waviness, the Rz of DFC was found to increase significantly after the toothbrushing experiment, but had much lesser waviness compared with PMMA in the waviness analysis and SEM imaging results (Figure 8). Conversely, PMMA had a lower Rz than did FMLB, NXT, PLC, and DFC, but the waviness analysis and SEM imaging results indicated that the waviness increased significantly due to concave abrasion as the vertical loss increased after brushing. This was consistent with the comparison between the wear levels of CAD/CAM blocks performed by Choi et al. [37], which indicated that PMMA exhibited a greater volume and weight losses than did DFC and PICN. Regarding the evaluated 3D printed materials, there was no significant difference in the amount of vertical loss, suggesting that they can be used as temporary restorative materials for 3D printing crowns and bridge resins.

The VHN test can be useful for evaluating the properties of composite materials related to wear resistance [25,38,39]. Dupriez et al. [40] suggested that knowledge of the surface hardness of materials is essential for predicting wear resistance. The VHNs of FMLB, NXT, PLC, and PMMA were indicated to be similar, while DFC had a larger VHN, and this was highest in PICN. This result was consistent with the low surface waviness of DFC and PICN found in the surface waviness and SEM imaging results. These differences in surface hardness between materials can be explained by the internal composition of the materials [41]. The inorganic fillers distributed in the DFC protect its matrix, and the nano-sized fillers and narrow interparticle spacing improve the surface hardness. The 3D interconnected double-ceramic network structure of PICN results in better physical properties than conventional composites [6,42]. In contrast, acrylic resins may have smaller mechanical strength due to their relatively low double-bond conversion [43]. Goujat et al. [44] and Kim et al. [45] evaluated the surface hardness of CAD/CAM materials and 3D printed resins. Their results indicated that PICN had the largest surface hardness trend, followed by DFC. The surface hardness of 3D printed resin was relatively low, which was similar to the trend found in the present study. As a characteristic finding, surface hardness measurements after artificial toothbrushing indicated that the surface hardness of FMLB and NXT somewhat increased. This may be due to the additional polymerization of the printed resin that occurred during the experiment as the daily light exposure time increased. Following the same principle, it can be interpreted that the previous finding of the VHN increasing may have been due to the increased polymerization time of the NXT specimen [45]. It may also be due to the oxygen-inhibiting layer on the surface of the specimen, which appears before artificial brushing. Studer et al. [46] evaluated the conversion degree of a photopolymerized resin exposed to oxygen, and found that the resin polymerization slowed and the conversion degree decreased as the sample came into contact with atmospheric oxygen before photocuring. These layers can affect the surface properties of the photopolymerization resin, such as its strength and scratch resistance [46]. This may be due to the layer being removed by artificial toothbrushing, and surface hardness increasing accordingly.

The color stability of dental restorative material plays a decisive role in material selection, especially for anterior restorations, and ΔE values larger than 2.25 are considered clinically observable [47,48]. These materials must therefore have color stability that can withstand microscopic abrasion caused by toothbrushing and constant moisture exposure [49]. In the present study, color changes in the materials between, before, and after artificial toothbrushing were recorded in CIE L* a* b* color space using a spectrophotometer. Spectrophotometry is the most accurate and commonly recommended method because it can objectively and quantitatively evaluate color changes of a material [48]. The present measurements indicated that DFC had a significantly high color difference of ΔE = 2.4 ± 0.9, but the ΔE values of the remaining groups were lower than the clinical limit of 2.25, indicating that they were within the perceptual threshold. FMLB and PLC had significantly lower color differences, and the NXT, PMMA, and PICN groups had similar color changes. Artificial toothbrushing causes surface erosion, which can release components that affect the color tone of the resin and increase surface deterioration [50]. Resin water absorption can also increase pigment migration or adhesion [51,52]. This suggests that the increased water absorption of the 3D printed resin may have affected the color changes observed. This was consistent with Yuan et al. [1] finding that color differences were within the perceptual threshold after 50,000 toothbrushing cycles on CAD/CAM lithium disilicate and zirconia specimens. Haynie et al. [53] immersed 3D printed resin samples in distilled water to test discoloration, and found that the color change was within the critical limit range. However, if coloring factors do occur, different results may appear. A previous study applied foods with coloring elements (e.g., coffee, grape juice, and curry) to CAD/CAM blocks and 3D printed resin, and found that the color change of 3D printed resin was larger than that of the CAD/CAM material [48]. Regarding 3D printed resin, additional research is therefore needed on color stability based on parameters, such as discoloration source and postcuring time.

In this study, hydrothermal aging treatment was applied to specimens to simulate temperature changes in the oral environment and to determine their effect on flexural strength. The flexural strength differed between the groups without any treatment, and was significantly larger in the 3D printed resin group than for DFC, PMMA, and PICN. Differences in the flexural strength of materials may be attributed to the polymerization process of the resin and the monomer composition and chemical composition variables [54], which affect flexural strength and mechanical properties [55,56]. Differences in mechanical properties may also appear due to differences in the densities of the materials [57]. Simoneti et al. [58] compared the flexural strengths of 3D printed resin and dental CAD/CAM milling material. They found that the flexural strength of the 3D printed material was significantly larger than those of PMMA, DFC, and PICN, which was consistent with the trend found in the present study. Goujat et al. [44] also compared the flexural strengths of CAD/CAM materials, and found that DFC had slightly larger flexural strength than PICN, which was similarly consistent with the results of the present study.

The NXT, PMMA, DFC, and PICN groups were affected by hydrothermal aging, with their flexural strengths tending to decrease. This can be attributed to surface deterioration and water absorption occurring during the high-temperature and high-pressure hydrothermal aging process. When the resin expands at high temperatures and concurrently absorbs water, water may penetrate the resin matrix and soften the polymer [59]. This may also be caused by absorbed water hydrolyzing the interfacial silane coupling agent, which provides chemical bonding with the resin structure and filler, resulting in decreased flexural strength [60]. Berli et al. [61] found that flexural strength decreased after hydrothermal aging when comparing the mechanical properties of milling materials and 3D printed resin, which was consistent with the results of the present study. The flexural strength of PICN before aging indicated similar values to the flexural strengths obtained by Argyrou et al. and Kurtulmus-Yilmaz et al. [62,63]. However, its flexural strength decreased significantly to 73.6 ± 8.0 MPa after hydrothermal aging. The 3D printed resin group exhibited a significantly larger flexural strength than did DFC, PMMA, and PICN, and there was no significant difference in the flexural strength of FMLB after hydrothermal aging. This suggests that 3D printed resin can be used instead of milling materials.

In this in vitro study, the changes in the mechanical properties and color of materials were observed after 20,000 artificial toothbrushing cycles, and flexural strengths after hydrothermal aging were also evaluated. The test results of the milling material are consistent with the results of previous studies, indicating that the results obtained are reliable. Therefore, the experimental results on 3D printed resin materials can serve as a reference to supplement the lack of knowledge about the properties of materials, suggesting that that the 3D printed materials have comparable mechanical and optical properties that could be a substitute to the current milling materials. However, this study had several limitations. Experimental artificial toothbrushing could not simulate various aspects of the real environment including changes in oral pH, chewing force, and the presence of bacteria and saliva. The results would reflect the clinical environment better if the oral biological environment is simulated more accurately, and factors such as the mastication force and mastication frequency were also included. The specimens used in this study had a flat shape and so did not reflect actual tooth curvature. The abrasion test results may indicate different characteristics than for the curved contours of actual teeth, so verification through further clinical studies will be required. The types of resins used in this study were also relatively restricted because the evaluation was conducted using materials that represented the experimental groups. Many companies now manufacture and sell various 3D printing and milling materials, and different results for mechanical properties and color stability may appear in similar experiments depending on the composition of the resin used and the color change source; the effects of various parameters should therefore be evaluated in the future.

The 3D printed material evaluated in this study exhibited mechanical and optical properties comparable to the current milling materials, and was observed to be clinically stable. 3D printed prostheses thus could be used for similar purposes to fabricate prostheses using prefabricated milling materials. The 3D printer is being supplied at a relatively lower price, and it can overcome the disadvantages of the milling method, which is a significant consumption of materials and milling burs [9,13]. On the other hand, in the case of milling, cutting efficiency may decrease due to bur ware, which may affect accuracy and increase maintenance costs [11,64]. Furthermore, various applications of 3D printing such as implants [65], anatomical models [66], and maxillofacial reconstruction [67,68] have been reported in previous studies because complex structures can be easily fabricated with 3D printing [5]. Therefore, the clinical use of 3D printing technology will increase productivity and provide a more convenient way to fabricate provisional restorations [11]. However, for a wider application of the 3D printing technology in dental care, it will have to be verified through additional studies to examine compressive, tensile, shear, and fatigue strength along with solubility and permeability. Different results may appear depending on the type of toothbrush or toothpaste used. Therefore, physical properties of the 3D printed resin materials with respect to many factors should be studied in the future. Also, standardized protocols and studies that are more consistent with the real oral environment are therefore needed in the future.

## 5. Conclusions

The use of resin materials in the 3D printing manufacturing for making a dental restoration is worthy of study in a novel way. The purpose of this study was to investigate the mechanical properties and color stability of the 3D printed resin materials compared with the milling materials after artificial toothbrushing and hydrothermal aging. Within the limits of this in vitro study, the mechanical and optical properties of the 3D printed resin materials were in a range comparable to the milling materials. The 3D printing method can overcome the disadvantage of the milling method including significant consumption of material and inaccuracy resulting from bur wear. Consequently, 3D printed resin materials may offer better advantages as an alternative to milling materials for fabrication of temporary dental restorations.

## Figures and Tables

**Table 1:** Characteristics of 3D printing and CAD/CAM blocks used in this study (The composition was written followed by the manufacturer’s information).

Type	Product	Code	Composition	Manufacturer
3D printed resin	Denture teeth A2;	FMLB	Methacrylate monomer, diurethane dimethacrylate, propylidynetrimethyl trimethacrylate	Formlabs, Sommerville, MA, USA
3D printed resin	NextDent C&B	NXT	>90% Methacrylic oligomers, methacrylate monomer, <3% phosphine oxides, pigment	NextDent, Soesterburg, the Netherlands
PLC-based CAD/CAM material	PLC ‡	PLC	PLC (88 wt%), inorganic filler (2 wt%), nano silica (8 wt%), glass fiber additive (1 wt% alkoxysilane)	Line Dental Lab, Seoul, Korea
PMMA-based CAD/CAM material	VIPI BLOCK	PMMA	Highly cross-linked PMMA	Dental VIPI, VIPI, São Paulo, Brazil
Resin nano ceramic CAD/CAM material (DFC)	MAZIC Duro	DFC	20 wt% reinforced matrix, 80 wt% ceramic nanofillers	Vericom, Chuncheon, Korea
PICN CAD/CAM material	VITA ENAMIC	PICN	86 wt% feldspathic-based ceramic network, 14 wt% acrylate polymer network (infiltrated into feldspathic-based ceramic network)	VITA Zahnfabrik, Bad Säckingen, Germany

‡ The PLC CAD/CAM disk used in this study was a prototype that had not yet been marketed.

## Data Availability

The data presented in this study are available on request from the corresponding author.

## Supplementary Materials

The following are available online at https://www.mdpi.com/article/10.3390/ma14206207/s1, Table S1: Surface roughness and surface waviness before and after toothbrushing, Table S2: Vickers hardness results of six materials before and after toothbrushing, Table S3: color difference results of six materials, Table S4: Flexural strength results of six materials before and after toothbrushing.
