# Supplementary material for: Effects of Artificial Tooth Brushing and Hydrothermal Aging on the Mechanical Properties and Color Stability of Dental 3D Printed and CAD/CAM Materials
Source: Materials (Basel). 2021 Oct 19;14(20):6207. doi: 10.3390/ma14206207 (PMC8540203; doi:10.3390/ma14206207)
Supplement: Supplementary file 1 [file materials-14-06207-s001.zip › materials-1382402-supplementary.pdf]

Supplementary Table S1. Surface roughness (Ra, Rz) and surfcae waviness (Wa, Wa) in μm (data are mean and standard-deviation values) before and after artificial toothbrushing. A significant difference between before and after artificial toothbrushing is indicated by different lower-case letters ($p<0.05$).

		FMLB	NXT	PLC	PMMA	DFC	PICN
Ra	Pre	0.17±0.02[a]	0.16±0.03[a]	0.01±0.00[a]	0.02±0.01[a]	0.01±0.00[a]	0.03±0.01
	Post	0.17±0.05[a]	0.18±0.02[b]	0.07±0.03[b]	0.08±0.05[b]	0.16±0.02[b]	0.04±0.01
Rz	Pre	0.92±0.11[a]	0.83±0.13[a]	0.07±0.02[a]	0.19±0.06[a]	0.05±0.02[a]	0.22±0.08
	Post	0.88±0.24[a]	0.89±0.07[b]	0.43±0.13[b]	0.43±0.23[b]	0.84±0.11[b]	0.29±0.04
Wa	Pre	2.64±5.11[a]	2.85±2.95[a]	0.43±0.13[a]	0.43±0.23[a]	0.84±0.11[a]	0.29±0.04
	Post	4.67±4.28[a]	4.04±3.05[a]	2.13±0.80[b]	8.67±4.03[b]	1.95±0.63[b]	0.44±0.13
Wz	Pre	0.29±0.23[a]	0.38±0.10[a]	0.06±0.01[a]	0.08±0.02[a]	0.03±0.00[a]	0.14±0.04
	Post	0.62±0.18[b]	0.49±0.08[b]	0.22±0.06[b]	1.30±0.58[b]	0.32±0.04[b]	0.44±0.13

Supplementary Table S2. Vickers hardness (data are mean and standard-deviation values) of six materials. A significant difference between materials is indicated by different upper-case letters, and a significant difference between before and after artificial toothbrushing is indicated by different lower-case letters ($p<0.05$).

	FMLB	NXT	PLC	PMMA	DFC	PICN
Pre	9.1±0.4[A,a]	15.5±0.4[A,a]	16.5±0.9[A,a]	16.9±1.1[A,a]	94.1±6.3[B,a]	251.6±26.2[C,a]
Post	12.7±2.7[A,b]	16.1±6.1[A,b]	16.4±6.4[A,a]	16.3±6.3[A,a]	90.6±0.6[B,a]	235.5±22.8[C,a]

Supplementary Table S3. color difference results (data are mean and standard-deviation values) of six materials. A significant difference between materials is indicated by different lower-case letters ($p<0.05$).

	FMLB	NXT	PLC	PMMA	DFC	PICN
ΔE	1.0±0.6[a]	1.6±0.6[ab]	1.2±0.2[a]	1.4±0.7[ab]	2.3±1.0[b]	1.4±0.6[ab]

Supplementary Table S4. Flexural strength results (data are mean and standard-deviation values) in MPa before and after toothbrushing. Significant differences ($p<0.05$) between materials and between before and after hydrothermal aging are indicated by different upper-case letters and different lower-case letters, respectively.

	FMLB	NXT	PLC	PMMA	DFC	PICN
Pre	139.4±40.5[BC,a]	163.9±14.0[C,b]	155.2±23.6[C,a]	108.0±8.1[A,b]	116.4±11.4[AB,b]	104.2±19.0[A,b]
Post	135.5±37.8[B,a]	147.2±15.7[B,a]	147.1±38.3[B,a]	78.5±7.3[A,a]	86.9±11.4[A,a]	73.6±8.0[A,a]
